# Improvement of low‐intensity long‐time running performance in rats by intake of glucosyl hesperidin

**DOI:** 10.14814/phy2.15413

**Published:** 2023-01-28

**Authors:** Suminori Nagayama, Kai Aoki, Shoichi Komine, Norie Arai, Shin Endo, Hajime Ohmori

**Affiliations:** ^1^ Graduate School of Comprehensive Human Sciences University of Tsukuba Tsukuba Ibaraki Japan; ^2^ Faculty of Medicine University of Tsukuba Tsukuba Ibaraki Japan; ^3^ Japan Society for the Promotion of Science Kojimachi Business Center Building Chiyoda‐ku Tokyo Japan; ^4^ Department of Acupuncture and Moxibustion, Faculty of Human Care Teikyo Heisei University Toshima‐ku Tokyo Japan; ^5^ Hayashibara Co., Ltd Okayama Japan; ^6^ University of Tsukuba Tsukuba Ibaraki Japan; ^7^ Department of Sports and Health Management, Faculty of Business Information Sciences Jobu University Isesaki Gunma Japan

**Keywords:** ergogenic aid, hesperidin, low‐intensity exercise, β‐oxidation

## Abstract

Recently, the use of ergogenic aids in sports by both athletes and fans has increased. Moreover, the overall demand for new ergogenic aids has increased. Hesperidin is a polyphenol that is useful for improving exercise performance by activating energy generation through β‐oxidation and oxidative phosphorylation in skeletal muscles. However, it is difficult to use this compound as an ergogenic aid because of its poor water solubility and low bioavailability. Glucosyl hesperidin is formed when one molecule of glucose is transferred to hesperidin via glycosyl‐transferase. It is 10,000× more soluble and has 3.7× higher bioavailability than hesperidin. In this study, we assessed whether continuous (14 days) intake of glucosyl hesperidin improves the aerobic exercise capacity of rats during long‐term acute exercise. Although glucosyl hesperidin intake did not improve the performance of high‐intensity running (30 m/min), we did observe improvement in low‐intensity running (15 m/min) (*p* < 0.05). We demonstrate that in sedentary rats, glucosyl hesperidin intake increased β‐oxidation and oxidative phosphorylation in the skeletal muscle (*p* < 0.05 and *p* < 0.01, respectively). Glucosyl hesperidin intake may have created a metabolic state useful for long‐term exercise. In conclusion, the continuous intake of glucosyl hesperidin improved the aerobic exercise capacity of rats during long‐term acute exercise.

## Graphical abstract

Glucosyl hesperidin is formed when one molecule of glucose is transferred to hesperidin via glycosyl‐transferase. It is 10,000 times; more soluble and has 3.7 times; higher bioavailability than hesperidin. #10;In this study, we assessed whether continuous intake of glucosyl hesperidin improves the aerobic exercise capacity of rats during long‐term acute exercise.

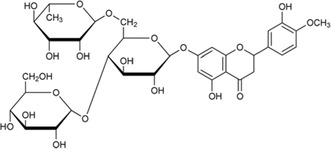

## 1 INTRODUCTION

Following recent advances in sports nutrition, the number of athletes who use nutritional supplements to improve exercise capacity and the effect of training, pre‐game conditioning, and facilitation of post‐training recovery from fatigue has increased (Maughan, 1999; Pipe & Ayotte, 2002). Especially in terms of improvement in aerobic exercise capacity, studies on the effect of a wide range of substances, such as functional elements, have reported that intake of medium‐chain fatty acids increases high‐intensity aerobic exercise capacity (Nosaka et al., 2009) and intake of caffeine extends the duration of aerobic exercise (Bell & McLellan, 2002).

Recent studies have investigated the effect of polyphenols on exercise performance. Plants produce polyphenols as secondary metabolites, which are ubiquitous in plant tissues (Duthie et al., 2003). Several studies have reported that polyphenols serve as effective sports supplements by suppressing exercise‐induced oxidative stress, inflammation, and myalgia, facilitating muscular glucose metabolism, and improving exercise capacity (Belviranli et al., 2012; Cases et al., 2017; Pereira‐Caro et al., 2017; Vlavcheski et al., 2017). Hespeiridin (Hes), a polyphenol abundantly present in the peel of citrus fruits, is gaining attention in this field. In pharmacokinetics, Hes is converted into physiologically active hesperetin (Hest) in the intestine and is subsequently absorbed in this form. In rats undergoing high‐intensity training, Hes eliminates reactive oxygen species (ROS) by acting as a free radical scavenger and activating Catalase, an antioxidative enzyme in vivo (Estruel‐Amades et al., 2019). Therefore, Hes suppresses exercise‐induced ROS and oxidative stress, and improvement was noted in the running distance achieved until exhaustion and in the effect of running‐based training (Estruel‐Amades et al., 2019). In another study involving the administration of a single dose of Hes to cyclists practicing habitual exercise, anaerobic splint capacity was improved (Martínez‐Noguera et al., 2019). In an in vitro study involving the addition of Hes to human myotube cells, an increase was noted not only in the level of adenosine triphosphate (ATP) with‐in the muscle cells, but also in oxidative phosphorylation (OXPHOS), peroxisome proliferator‐activated receptor gamma coactivator‐1 alpha (PGC1‐α), and the expression of mitochondrial genes (Biesemann et al., 2018). However, there are many hurdles to the commercialization of Hes as an ergogenic aid for general use owing to its low water solubility and bioavailability.

Glucosyl hesperidin (GHes) is formed by the attachment of a glucose molecule to Hes. It has been reported to have approximately 10,000 times higher water solubility and 3.7 times higher bioavailability than Hes alone (Yamada et al., 2006). In addition, GHes is degraded into glucose and Hes by enzymatic activity in the small intestine and subsequently manifests a pharmacokinetic profile similar to that of Hes (Yamada et al., 2006). Therefore, GHes can be commercialized as a supplement more easily than Hes, and the ergogenic effect of the physiological activity of Hes may be elevated by GHes. The efficacy of GHes has so far been confined to studies using animal models with pathological conditions, such as high‐fat diet‐induced obesity and spontaneous onset hypertension, where it has been demonstrated that GHes improves fat metabolism and blood pressure and suppresses oxidative stress (Tomazini Gonçalves et al., 2018; Yamamoto et al., 2013). Studies have reported that continuous GHes intake in combination with physical training suppresses exercise‐induced oxidative stress and improves fat metabolism (de Oliveira et al., 2013; Tomazini Gonçalves et al., 2018). However, no study has reported its effect on exercise capacity.

The present study was conducted to evaluate the effect of continuous GHes intake on exercise capacity in non‐trained rats from two perspectives: (1) the duration of running until exhaustion during exercise at two intensity levels (high‐intensity running at 30 m/min and low‐intensity running at 15 mg/min) and (2) the effect of biological molecules related to exercise capacity.

## 2 MATERIALS AND METHODS

### 2.1 Animal treatment

This study was approved in advance by the Experimental Animal Ethics Committee (Approval No. 19–350) in accordance with the University of Tsukuba Guidelines on Animal Studies. A total of 35 eight‐week‐old male Wistar rats (Japan SLC, Shizuoka, Japan) that were free of abnormalities (e.g., injuries and sickness) during the study period were included in the analysis. The animals were housed individually in a controlled environment with a temperature of 20–26°C, 40%–60% relative humidity, and a 14:10 light: dark cycle (8:00–22:00 light). Animals were allowed free access to a standard chow diet (MF diet; Oriental Yeast Co., Tokyo, Japan). Distilled water was supplied as the drinking water. An aqueous GHes solution or control solution was provided during the intake period. Body weight and water intake were recorded daily from 8:00–12:00 during the intake period.

### 2.2 Supplement preparation

**FIGURE 1:**
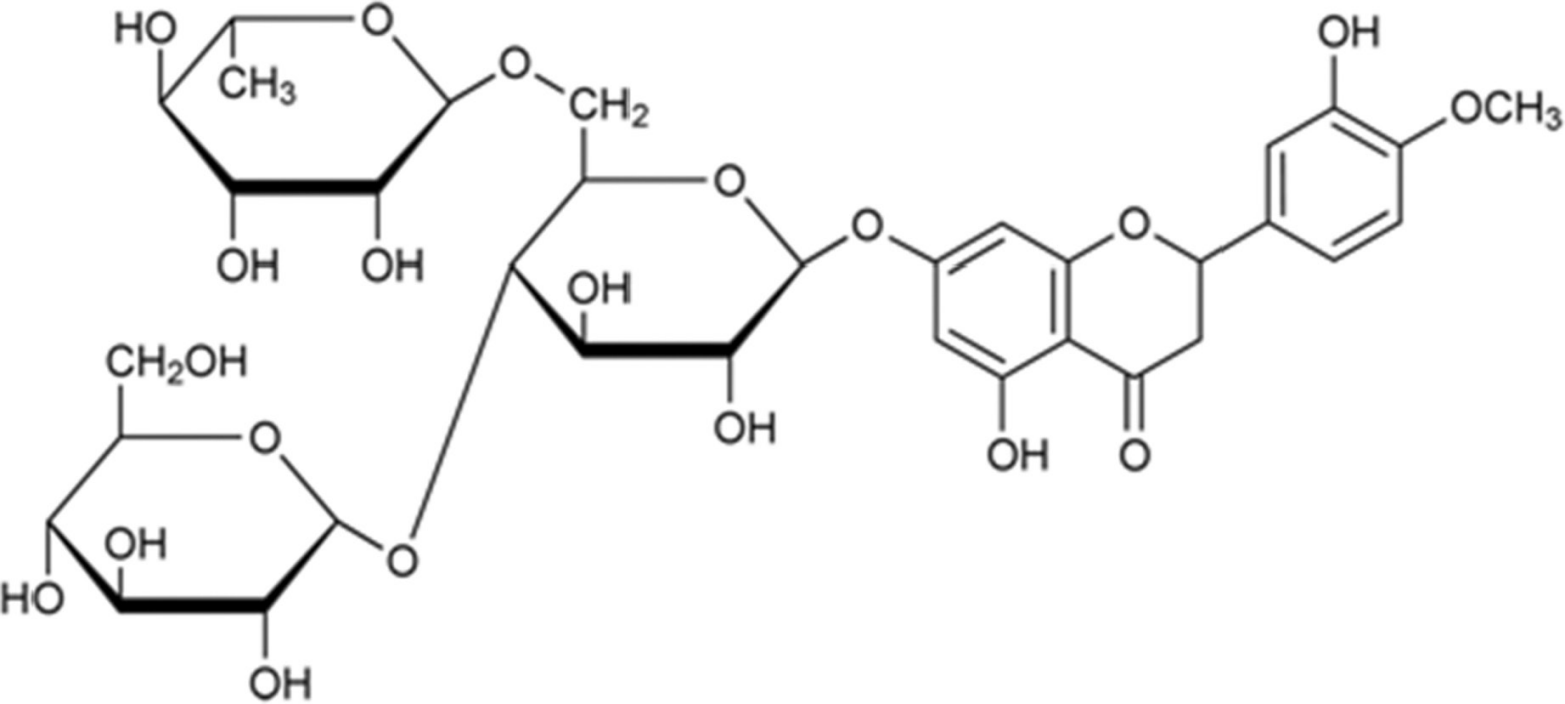
Molecular structure of glucosyl hesperidin.

Hayashibara Hesperidin^Ⓡ^ S‐Glucosyl hesperidin (Hayashibara, Okayama, Japan) was dissolved in distilled water to yield an aqueous GHes solution (the molecular structure is shown in Figure 1). This solution was placed into a water supply bottle in a volume adjusted based on the rat's body weight (BW) and water intake data on the day of intake to ensure a daily intake of approximately 50 mg/kg BW. This dosage was predicted to increase blood levels based on previous studies (Yamada et al., 2006). Each animal was allowed free access to water using this bottle. For the control aqueous solution, D(+)‐glucose (Fujifilm Wako Pure Chemical, Osaka, Japan) was dissolved in distilled water at a concentration allowing administration of approximately 11.7 mg/kg BW of glucose per day (equivalent to the amount of glucose contained in Hayashibara Hesperidin^Ⓡ^S‐Glucosyl Hesperidin 50 mg/kg BW; C34H44O20 = 772 g/mol; and C6H12O6 = 180 g/mol ⇒ 180/772 × 50 (mg) = 11.7 (mg)) on the basis of the rat's BW and water intake on the day of intake. This solution was placed in a water supply bottle, allowing the animals free access to water for drinking.

### 2.3 Grouping and performance testing

**FIGURE 2:**
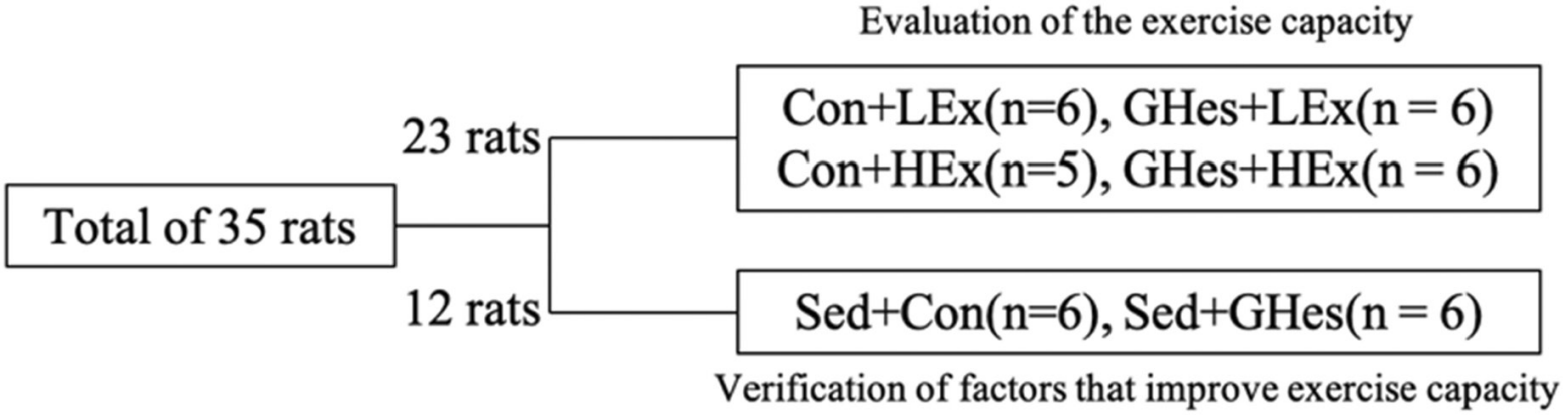
A schematic of the grouping. Con, Control; GHes, Glucosyl hesperidin; LEx, Low‐intensity exercise; HEx, High‐intensity exercise; Sed, Sedentary.

A schematic of this grouping is shown in Figure 2. A total of 35 rats were used in this study. Rats (*n* = 23) were randomly divided into GHes intake and control intake groups to evaluate exercise capacity. Each group was subdivided into a low‐intensity exercise group and high‐intensity exercise group. Therefore, the animals were divided into four groups: low‐intensity exercise group (GHes+LEx, *n* = 6), control intake low‐intensity exercise group (Con+LEx, *n* = 6), GHes intake high‐intensity exercise group (GHes+HEx, *n* = 6), and control intake high‐intensity exercise group (Con+HEx, *n* = 5). To explore factors involved in the improvement of exercise capacity, 12 additional rats were divid‐ed into two groups: sedentary control intake group (Sed + Con, *n* = 6) and sedentary GHes intake group (Sed + GHes, *n* = 6).

The running exercise experiment used a treadmill for small animals (FVRO, 4E9S‐6; Fuji Medical Service, Chiba, Japan). The rats practiced a trial run for five consecutive days, beginning 1 week before the exhaustion test, to familiarize themselves with running on the treadmill. The trial runs were performed between 8:00 and 12:00. After 14 days of GHes/control intake (5‐day trial run from Day 7 + 2‐day rest), each group was subjected to the exhaustion test. All animals were fasted for 3 h before starting the exhaustion test. After resting on the treadmill for 15 min, the animals were allowed to run for 3 min at a speed of 15 m/min (warm‐up). Thereafter, the animals were subjected to the main‐run task at a constant speed (15 m/min in the low‐intensity exercise group and 30 m/min in the high‐intensity exercise group) until exhaustion. The exhaustion test started between 8:00 and 10:00. After the exhaustion test, the animals were sacrificed without pain by means of an anesthetic overdose (pentobarbital, Kyoritsuseiyaku, Tokyo, Japan).

### 2.4 Sampling

To collect blood from the inferior vena cava, celiotomy was performed on each animal immediately after painless sacrifice with an anesthetic overdose. The collected blood was treated with an anticoagulant (heparin sodium) and immediately centrifuged at a low temperature (2000 × g at 4°C for 5 min) to extract plasma. The gastrocnemius muscle was collected as a tissue sample. Each sample was rapidly frozen in liquid nitrogen and stored at −80°C until analysis.

### 2.5 Blood glucose and lactate measurements

Blood was collected from the caudal vein of each rat immediately before and after exhaustion testing. Blood lactic acid levels were measured using a simplified blood lactic acid measuring device (LactatePro™ 2 LT‐1730; Arkray, Kyoto, Japan), and blood glucose levels were measured using a glucose self‐measuring device (One Touch Ultra^Ⓡ^; Johnson & Johnson, Tokyo, Japan).

### 2.6 Biochemical analysis of plasma

Plasma samples treated with heparin sodium were biochemically analyzed by the Oriental Yeast Nagahama Life Science Laboratory (Oriental Yeast Co., Ltd, Tokyo, Japan).

### 2.7 Total RNA extraction and RNA concentration measurement

Total RNA was extracted using Sepasol®‐RNA I Super G (Nacalai Tesque). The gastrocnemius muscle (100 mg) was combined with 1000 μl of Sepasol®‐RNA I Super G and the sample was homogenized with a bead‐type crusher (TissueLyser II, QUIAGEN, Germany). The crushed tissue was combined with chloroform and mixed by repeated inversion, followed by low‐temperature centrifugation (12,000 × g at 4°C for 15 min). The water layer (the upper layer of the sample) was transferred to another tube and combined with 500 μl 2‐propanol. This was followed by low‐temperature centrifugation (12,000 × g at 4°C for 10 min) to induce RNA sedimentation. The supernatant was discarded and the remainder was washed with 75% ethanol, dried for approximately 10 min, and dissolved in RNase‐free water (Thermo Fisher Scientific, USA) while being heated (65°C for 5 min). The concentration of the dissolved sample was measured using a NanoDrop spectrophotometer (Thermo Fisher Scientific). cDNA synthesis was performed using PrimeScript™ RT Master Mix (Perfect Real Time; Takara Bio, Shiga, Japan). After adjusting the concentration to 400 ng/tube, the total RNA sample was heated with a thermal cycler (Life Technologies, USA) for 15 min at 37°C, then 5 s at 85°C, followed by cooling to 4°C. The synthesized cDNA was diluted 1:10 with DNase/RNase‐free water to obtain a real‐time RT‐qPCR sample.

### 2.8 DNA extraction

The gastrocnemius muscle was homogenized in 1× SNET (20 mM Tris/HCl, pH 8.0, 5 mM EDTA, 400 mM NaCl, and 0.3% SDS) supplemented with proteinase K (Wako Pure Chemical, Osaka, Japan), followed by heating at 56°C for 1 h. Then, the mixture was combined with a 25:24:1 mixture of phenol, chloroform, and isoamyl alcohol (Nacalai Tesque, Kyoto, Japan), followed by intense agitation and centrifugation (14,000 × g at 20°C for 15 min). After centrifugation, the supernatant was combined with an equal volume of 2‐propanol, followed by centrifugation (14,000 × g at 20°C for 10 min), washed twice with 70% ethanol, and wind‐dried for 10 min. The sample was then combined with 100 μl of ultrapure water and dissolved while heated (65°C for 5 min). The DNA concentration in the dissolved sample was measured using a spectrophotometer (Thermo Fisher Scientific, USA). The sample was diluted with ultrapure water to obtain a DNA concentration of 10 ng/μl, yielding a real‐time RT‐qPCR sample for mitochondrial DNA analysis.

### 2.9 Real‐time RT‐qPCR

The KAPA SYBR® FAST qPCR Master Mix (2×) Rox Low Kit (NIPPON Genetics, Tokyo, Japan) served as the reaction fluid for PCR. RT‐qPCR analysis was performed using the QuantStudio real‐time PCR system (Thermo Fisher Scientific, USA). The Ct value obtained by real‐time RT‐qPCR analysis was used to calculate the expression level using the ΔCt technique (using the equation given below).

Expression level = 2^(housekeeping gene Ct value – target gene Ct value).

The primer sequences are shown in Table 1.

**TABLE 1:** List of primers used in this study

Gene name	F/R	Primer sequence
Catalase	Forward	5′‐CAGCGACCAGATGAAGCA‐3′
(*Catalase*)	Reverse	5′‐GGTCAGGACATCGGGTTTC‐3′
Superoxide dismutase 1	Forward	5′‐CCAGCGGATGAAGAGAGG‐3′
(*Sod1*)	Reverse	5′‐GGACACATTGGCCACACC‐3′
Glyceraldehyde‐3‐phosphate dehydrogenase	Forward	5′‐ATGACTCTACCCACGGCAAG‐3′
(*Gapdh*)	Reverse	5′‐GGAAGATGGTGATGGGTTTC‐3′
Genomic DNA (gDNA)	Forward	5′‐CAGTACTTTAAGTTGGAAACG‐3′
	Reverse	5′‐ATCAACATAATTGCAGAGC‐3′
Mitochondrial DNA (mtDNA)	Forward	5′‐TCCTCCGTGAAATCAACAACC‐3′
	Reverse	5′‐GGGAACGTATGGACGATGAAC‐3′

### 2.10 Total protein extraction and protein concentration measurement

The samples were stored at −80°C until being processed. Samples were combined with a mixture of RIPA buffer (1% NP‐40, 0.1% SDS, 20 mM Tris–HCl [pH 8.0], 5 mM EDTA, 150 mM NaCl), proteinase inhibitor (Complete Mini, Roche, Switzerland), and phosphatase inhibitor (PhosSTOP Phosphatase Inhibitor Cocktail, Roche, Switzerland), followed by homogenization with a bead‐type crusher (TissueLyser II, QUIAGEN, Germany). The homogenate was subjected to low‐temperature centrifugation (12,000 × g at 4°C for 15 min), and the supernatant was transferred into another tube to obtain the total protein fraction. The concentration of the extracted total protein fraction was measured using the TaKaRa BCA Protein Assay Kit (Takara Bio, Shiga, Japan). The absorbance at 562 nm was measured using a microplate reader (Varioskan, Thermo Fisher Scientific, USA).

### 2.11 SDS‐PAGE and immunoblotting

The Any kD™ Mini‐PROTEAN® TGX™ Precast Gel (Bio‐Rad, USA) was used for SDS‐PAGE. The protein sample was combined with sample buffer (0.25% Tris–HCl, 0.02% bromophenol blue, 8% SDS, 40% glycerol, and 10% 2‐mercaptoethanol), followed by heat treatment (45°C for 30 min). The polyacrylamide gel was placed in an electrophoresis tank (Mini‐PROTEAN® Tetra Cell, Bio‐Rad, USA), followed by the addition of an electrophoresis buffer (250 mM Tris, 1.92 M glycine, and 1% SDS). Then, 20 μg of each sample was added into the wells and electrophoresis was performed at a constant voltage (150 V) for 30 min or longer. Proteins were transferred onto a polyvinylidene fluoride (PVDF) membrane using a semi‐dry blotting device (Trans‐Blot Turbo™ Transfer System, Bio‐Rad, USA) and semi‐dry transfer kit (Trans‐Blot® Turbo™ Mini‐PVDF Transfer Pack, Bio‐Rad, USA). In accordance with the recommended protocol, transfer of 5–150 kDa proteins was carried out at 2.5 A and 25 V for 3 min and transfer of 150 kDa or larger proteins was conducted at 2.5 A and 25 V for 10 min. Target proteins were detected on the post‐transfer membrane. Re‐probing was conducted before the same membrane was used for another detection session. The antibodies used and their dilution ratios are listed in Table 2.

**TABLE 2:** List of antibodies used in this study

Product code	Protein name	Spiecies	Dilution
CST #5831S	AMPKα	Rabbit	1:1000
CST #2535S	p‐AMPKα (Thr172)	Rabbit	1:1000
Santa Cruz sc‐518038	PGC‐1α	Mouse	1:1000
Santa Cruz sc‐393070	CPT1	Mouse	1:1000
Santa Cruz sc‐377294	CPT2	Mouse	1:500
Santa Cruz sc‐32233	GAPDH	Mouse	1:3000
Abcam ab110413	OXPHOS	Mouse	1:8000
CST #7076	Anti‐mouse IgG, HRP‐linked antibody	Goat	1:5000 or 1:10000
CST #7074	Anti‐rabbit IgG, HRP‐linked antibody	Goat	1:5000 or 1:10000

### 2.12 Statistical analysis

All data are expressed as the mean ± standard deviation. Two way‐analysis of variance (ANOVA) was used for comparison followed by Tukey's post hoc test to evaluate significance between four groups. The unpaired Student's *t*‐test was used for comparison between the two groups. For all tests, the significance level was set at *p* < 0.05. Statistical analyses were performed using GraphPad Prism 8.4.3 for Mac (GraphPad Software, San Diego, CA, USA).

## 3 RESULTS

### 3.1 Changes in time until exhaustion

**FIGURE 3:**
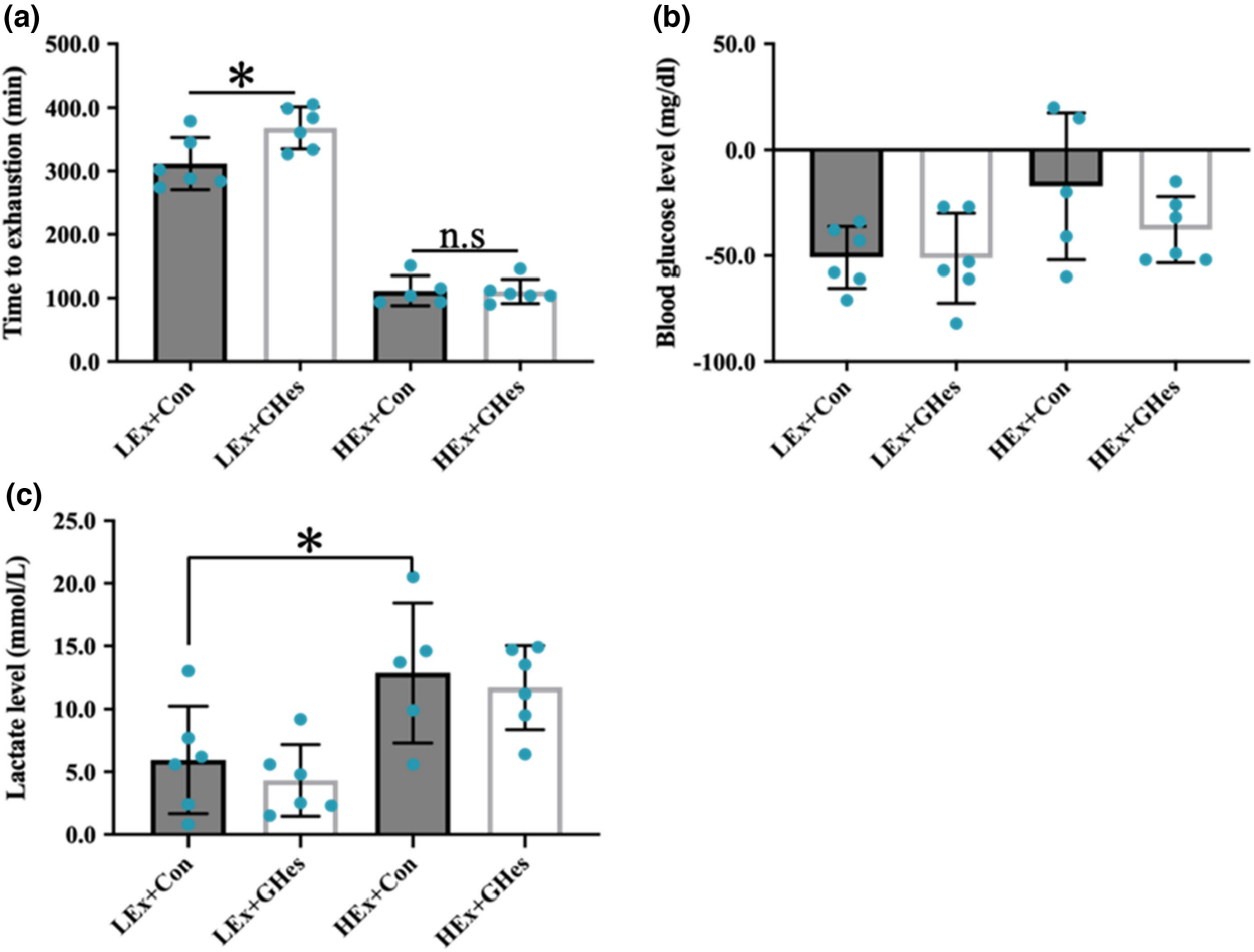
Effects of GHes supplementation on the exhaustion test in each exercise intensity group. (a) Time to exhaustion in LEx and HEx groups. (b) Blood glucose level in LEx and HEx groups. (c) Lactate level in LEx and HEx groups. (b) and (c) indicate the amount of change after the exhaustion test. Values indicate means ± *SD* (*n* = 5–6). Asterisk indicates the significant differences (*p < 0.05). Con, Control; GHes, Glucosyl hesperidin; LEx, Low‐intensity exercise; HEx, High‐intensity exercise.

In the low‐intensity exercise group, GHes intake resulted in significant extension of the time until exhaustion. In contrast, in the high‐intensity exercise group, GHes intake did not result in any significant change in the time until exhaustion (“all out” time) (Figure 3a).

### 3.2 Changes in blood lactic acid and glucose levels

GHes intake did not result in any significant change in blood glucose levels in the low‐intensity (vs. LEx + Con) or the high‐intensity exercise groups (vs. HEx+Con) (Figure 3‐b). The blood lactic acid level was significantly elevated in the high‐intensity groups in control intake group (Figure 3c).

### 3.3 Changes in plasma composition

Plasma levels of non‐esterified fatty acid (NEFA) and lipase (Lip) were significantly elevated by GHes intake (Table 3).

**TABLE 3:** Plasma biochemical data in Sed group

Blood marker	Sed + Con	Sed + GHes	*p*
TP (g/dl)	6.4 ± 0.1	6.4 ± 0.1	1.00
ALB (g/dl)	4.5 ± 0.1	4.6 ± 0.1	0.117
T‐CHO (mg/dl)	56.0 ± 2.2	54.3 ± 1.0	0.511
F‐CHO (mg/dl)	16.3 ± 0.3	16.5 ± 0.6	0.804
TG (mg/dl)	119.8 ± 17.4	134.5 ± 27.1	0.659
NEFA (μEq/L)	250.5 ± 18.1	309.7 ± 14.9	0.030*
GLU (mg/dl)	252.7 ± 12.2	238.0 ± 12.2	0.334
Lip (U/L)	6.5 ± 0.5	7.0 ± 0.0	0.049*

* Indicate significant differences compared with the control group (Con; *p < 0.05).

Abbreviations: ALB, Albumin; F‐CHO, Free Cholesterol; GLU, Glucose; Lip, Lipase; NEFA, Non‐esterified fatty acid; T‐CHO, Total Cholesterol; TG, Triglyceride; TP, Total protein.

### 3.4 Changes in anti‐oxidative function in vivo

**FIGURE 4:**
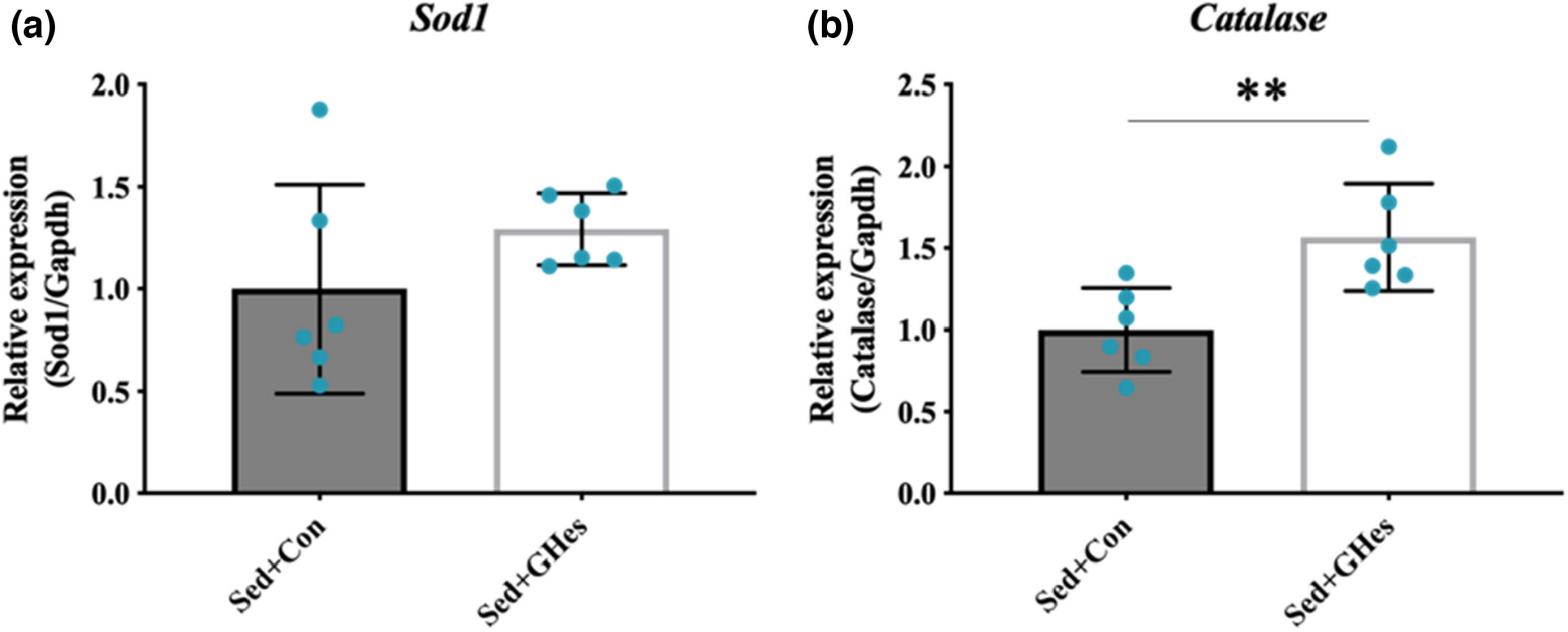
Effects of GHes supplementation on the expression levels of genes encoding antioxidant enzymes. (a) mRNA expression of the Sod1 gene in the Sed group. (b) mRNA expression of the Catalase gene in the Sed group. Sod1; Superoxide dismutase 1. Values indicate means ± *SD* (*n* = 6). Asterisks indicate significant differences relative to the control group (Con; **p < 0.01). Con, Control; GHes, Glucosyl hesperidin; Sed, Sedentary.

GHes intake did not cause any significant changes in the expression of Sod1, while expression of Catalase was significantly elevated (Figure 4b).

### 3.5 Changes in β‐oxidation‐related factors

**FIGURE 5:**
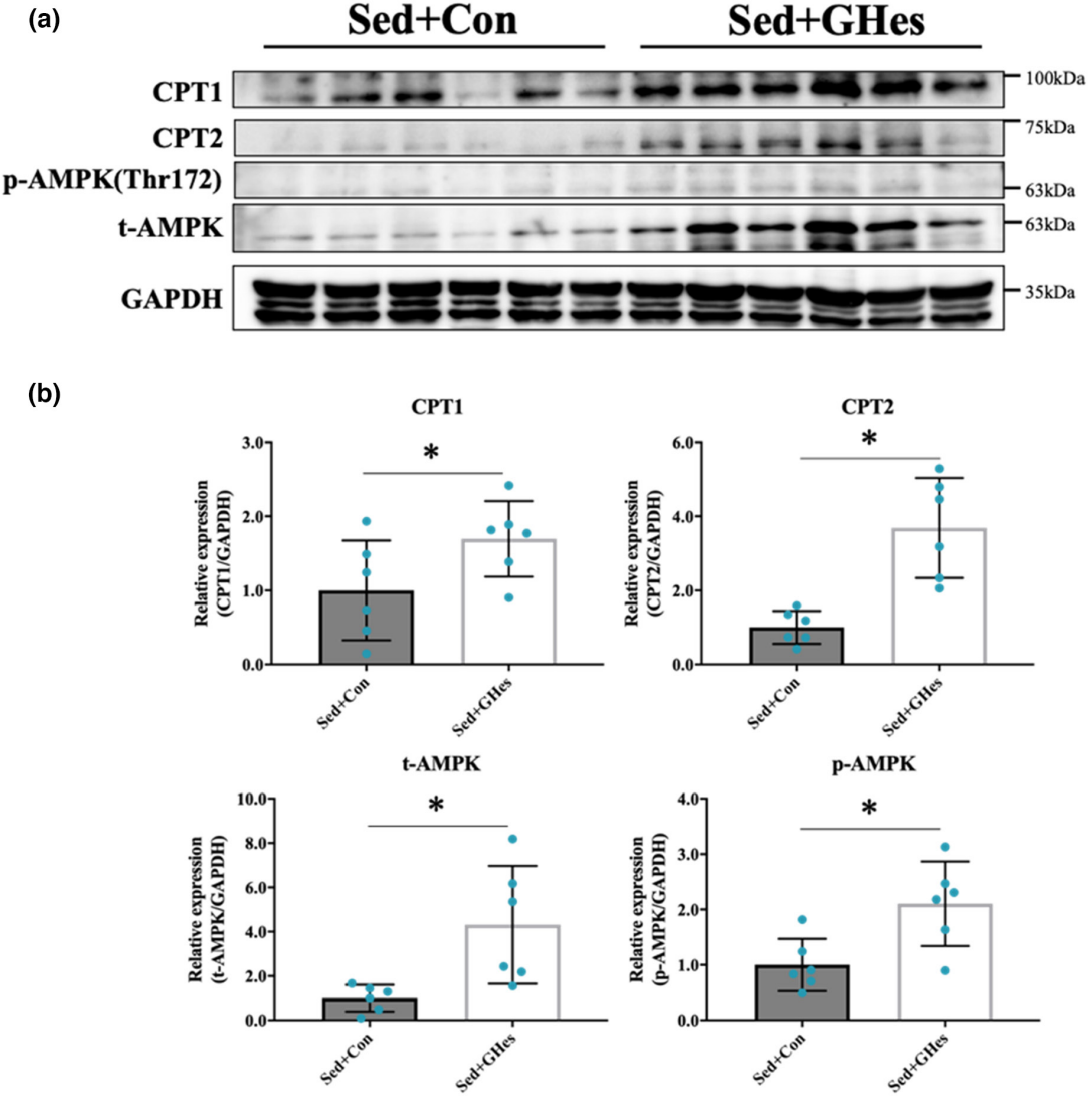
Effects of GHes supplementation on protein levels of β‐oxidation‐related fac‐tors. (a) Western blot of β‐oxidation‐related factors of rat gastrocnemius muscle samples. (b) Quantification of western blots. AMPK, AMP‐activated protein kinase (t, total; p, phosphorylated); CPT, Carnitine palmitoyl transferase; GAPDH, Glyceraldehyde‐3‐phosphate dehydrogenase. Values indicate means ± *SD* (*n* = 6). Asterisks indicate significant differences relative to the control group (Con; *p < 0.05). Con, Control; GHes, Glucosyl hesperidin; Sed, Sedentary.

GHes intake resulted in significant elevation of the levels of β‐oxidation‐related proteins (t‐AMPK, p‐AMPK, CPT1, and CPT2) (Figure 5).

### 3.6 Changes in mitochondria‐related factors

**FIGURE 6:**
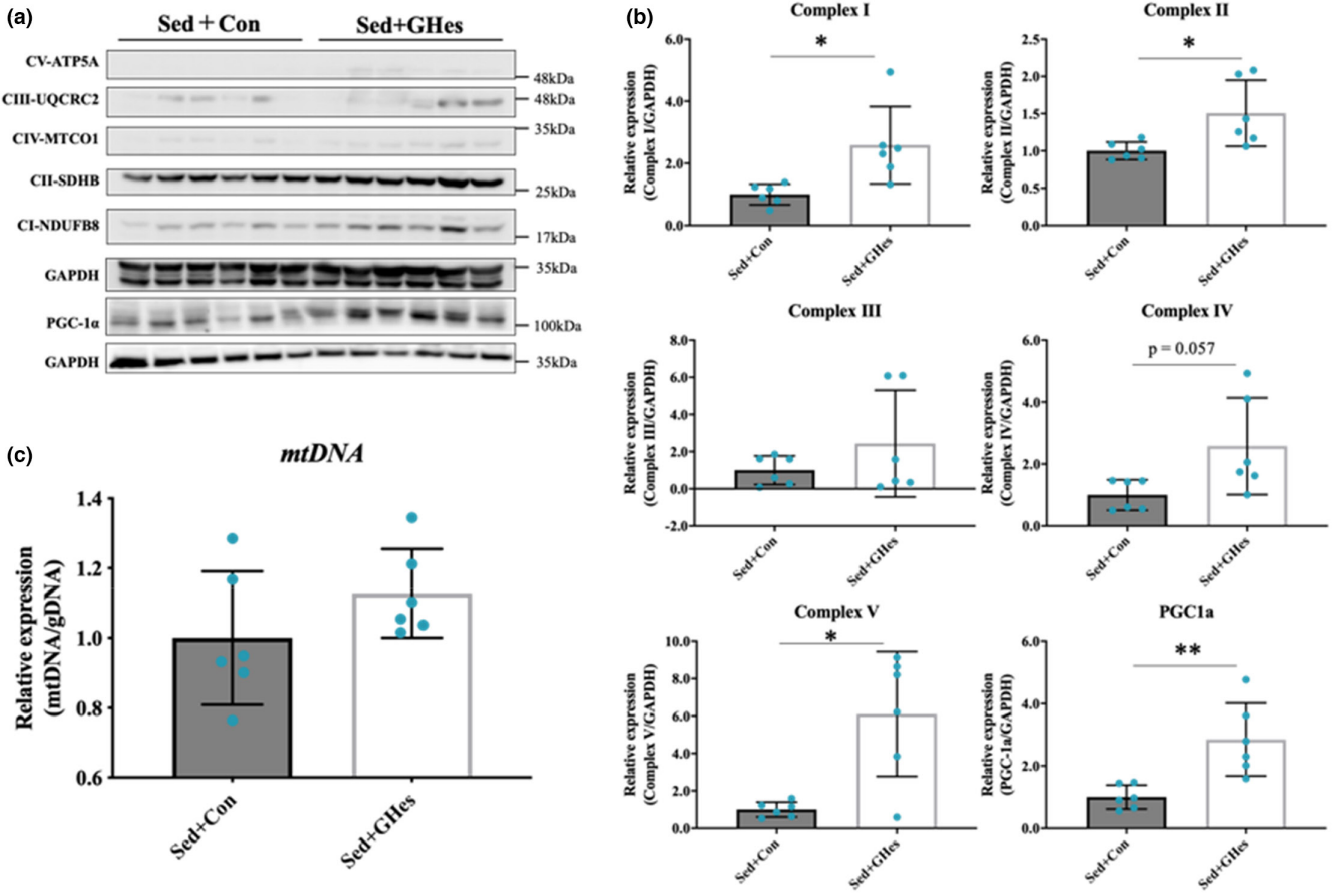
Effects of GHes supplementation on expression levels of genes and proteins controlling mitochondrial biogenesis and function related factors. (a) Western blot of mitochondrial biogenesis‐ and function‐related factors of rat gastrocnemius samples. (b) Quantification of western blot. (c) Quantification of mtDNA copy number in the Sed group. Complex I; NADH:quinone oxidoreductase, Complex II; Succinate dehydrogenase B, Complex III; Ubiquinol‐cytochrome‐c reductase, Complex IV; Cytochrome c oxidase, Complex V; ATP synthase subunits 5A, PGC‐1α; Peroxisome proliferator‐activated receptor gamma coactivator‐1α. Values indicate means ± *SD* (*n* = 6). Asterisks indicate significant differences relative to the control group (Con; *p < 0.05, **p < 0.01). Con, Control; GHes, Glucosyl hesperidin; Sed, Sedentary.

GHes intake resulted in significant elevation in the levels of OXPHOS complexes (CI‐NDUFB8, CII‐SDHB, and CV‐ATP5A) (Figure 6). There was no change in the mitochondrial DNA levels following GHes intake. PGC1‐α levels were significantly elevated by GHes intake (Figure 6).

## 4 DISCUSSION

The present study was designed to examine whether continuous GHes intake would improve aerobic exercise capacity and to analyze the changes related to such an effect in detail in vivo. First, the influence of GHes intake on the duration of running until exhaustion was evaluated in low‐intensity and high‐intensity running tests. The extension of running duration was observed only in the low‐intensity exercise group. Molecular biology components related to this outcome were explored through a detailed analysis of changes following GHes intake at rest.

We examined the influence of GHes intake on exercise capacity. GHes intake did not change the blood glucose or lactic acid levels; however, the high‐intensity exercise group showed a significant elevation in blood lactic acid levels compared to the low‐intensity exercise group (Figure 3). A previous study has demonstrated a marked elevation in blood lactic acid levels by exercise of lactate threshold or higher intensity, resulting in changes in metabolic responses in vivo (Soya et al., 2007). Therefore, we suggest that the exercise intensity for the low‐ and high‐intensity exercise groups in the present study was appropriate to examine the effects of GHes intake. Interestingly, the high‐intensity exercise group did not exhibit an extended duration of running until exhaustion (Figure 3). Oxidative stress is known to limit exercise capacity. It has been reported that the degree of exercise‐induced oxidative stress varies depending on the exercise intensity (Pyne et al., 2000). Exercise‐induced excessive ROS formation can arise following unfamiliar exercise or long‐lasting, high‐intensity exercise. Excessive ROS levels can induce muscle damage, resulting in a marked reduction in function. In contrast to our results, a previous study using a trained rat model reported that Hes intake elevated the activity of the antioxidant enzyme Catalase and improved exercise capacity during high‐intensity exercise (Estruel‐Amades et al., 2019). Muscles adapt to training, and thus, become acclimated to exercise. We observed that Catalase expression was increased by GHes intake (Figure 4), but this did not lead to an increase in exercise capacity during high‐intensity exercise, which may be because this study used non‐trained rats. Therefore, the influence of GHes on high‐intensity exercise should be studied further using trained animal models.

The duration of running was extended in the low‐intensity exercise group following GHes intake (Figure 3). To the best of our knowledge, this is the first study demonstrating an improvement in exercise capacity achieved by GHes intake, a possible reason for which could be the enhancement of β‐oxidation (an energy production system using fatty acids). The intake of GHes at rest resulted in plasma Lip activation and increased NEFA levels (Table 3). NEFAs are formed from triglycerides (TGs) contained in adipose tissues and lipoproteins, and are regulated by the activity of various lipases, such as lipoprotein lipase (LPL) and hormone‐sensitive lipase (HSL).

In the present study, it is likely that GHes intake activated Lip, resulting in stimulation of TG catalysis and elevation of plasma NEFA levels. A previous study demonstrated that LPL activation in skeletal muscles is mediated by adenosine monophosphate (AMP)‐activated protein kinase (AMPK) activation (Sasaki et al., 2014). There was no significant difference in the skeletal muscle AMPK phosphorylation level associated with GHes intake, but both total AMPK and phospho‐AMPK levels were increased (Figure 5). These results suggest that the increase in total AMPK levels resulted in an increase in total phosphorylated AMPK levels, eventually leading to the elevated AMPK signaling, which is a master regulator of fat metabolism. In addition, this study demonstrated increased expression of carnitine palmitoyl transferase (CPT) 1 and CPT2 in the sedentary group after being given GHes (Figures 5). This result is consistent with the findings of a previous study on obese rats (Mitsuzumi, 2011), but is the first report in a healthy animal model. CPT1 and CPT2 are rate‐limiting enzymes for β‐oxidation, and the increase in their expressions suggests enhancement of β‐oxidation and an increase in energy production arising from it (Adeva‐Andany et al., 2019).

The enhancement of β‐oxidation by the increased expressions of AMPK and CPT suggests that GHes intake affects the mitochondria metabolism, which is a source of energy produced by β‐oxidation. Therefore, we measured mitochondria‐related factors. The expression levels of PGC1‐α (a master regulator of mitochondrial biosynthesis) and OXPHOS complexes (C‐I, ‐IV, and ‐V; indicators of mitochondrial components) were significantly increased by GHes intake (Figure 6). In a study by Biesemann et al., Hes increased mitochondrial markers, including expression of PGC1‐α and level of ATP in human myotube cells (Biesemann et al., 2018). These findings are supported by the results of the present study regarding the influence of GHes intake. As AMPK activation and PGC1‐α stimulate mitochondrial biosynthesis (O'Neill et al., 2011; Short et al., 2005), we anticipated an increase in the number of mitochondria; however, no increase in mitochondrial DNA was found (Figure 6). Therefore, 2‐week GHes intake did not affect the number of mitochondria, but increased the expression of mitochondrial proteins. The Biesemann et al. study involved a 6‐week Hes intake by aged mice, which are important differences from the present study in terms of dosing period, dosing form, and animal conditions (Biesemann et al., 2018). This most likely explains why the increase was confined to the mitochondrial components. The dosing period during this study was 2 weeks, which may not be enough time to induce an increase in the number of mitochondria. However, an increase in the expressions of AMPK and PGC1‐α (factors involved in mitochondrial biosynthesis) suggests that long‐term GHes intake may contribute to an increase in the number of mitochondria. Overall, the administration of GHes to resting rats in this study may have created a metabolic state beneficial to long‐term exercise and thus contributed to the extended time to exhaustion.

This study has some limitations. Firstly, we used an untrained rat model, which may have prevented maximization of the effects of GHes. Training leads to various adaptations in the body. Some of these factors, such as antioxidant function and muscle endurance, are related to exercise performance. In the non‐training model of this study, it is possible that the exercise performance did not improve because of the inability of the muscles to withstand the intensity of high‐intensity exercise. Therefore, it is also necessary to examine the effect of GHes in a training model with similar dosing concentrations in the future. Secondly, the data on exercise capacity in this study was based on running time only. Exercise capacity includes muscle strength, muscle fatigue tolerance, and many other factors in addition to running time. In future research, in vivo grip strength and rotor rod tests and ex vivo muscle tension tests using isolated muscles will further clarify the effects of GHes on exercise capacity. Thirdly, the data supporting the mechanism of enhanced exercise capacity is limited to protein expression and gene expression analyses. By measuring the enzymatic activity of catalase and the activity of the mitochondrial respiratory chain, in addition to protein and gene expression, it will be possible to provide more robust evidence for functional improvement in the future. Finally, the present results are limited to healthy male rats. This study did not consider whether GHes has a similar effect on locomotor performance in female, middle‐aged, or old rats. Hence, further studies in animal models and human subjects are needed to determine whether GHes is effective in a wider range of subjects.

## 5 CONCLUSIONS

The present study demonstrated for the first time that a 2‐week GHes intake by rats improved the performance of low‐intensity running. Improved fat metabolism through β‐oxidation appears to be, at least in part, the mechanism for this effect from the analysis of resting rat intake of GHes. In addition, a concurrent increase in the expressions of OXPHOS complexes (mitochondrial components) was observed. This fundamental study demonstrated the effect of GHes as an ergogenic aid, which is of great interest to the field of exercise nutrition. It will be valuable to evaluate the effect of GHes on the exercise capacity of humans in a similar manner.

## AUTHOR CONTRIBUTIONS

Methodology, N. S., K. A., S. K., N. A., S. E., and H. O.; Validation, N. S., and K. A.; Formal analysis, N. S. and K. A.; Investigation, N. S., K. A., S. K., and H. O.; Writing—original draft preparation, N. S. and K. A.; Writing‐review and editing, N. S., K. A., S. K., and H. O.; Visualization, N. S., and K. A.; Supervision, S. K., N. A., S. E., and H. O.; Project administration, project administration; funding acquisition, S. K. and H. O. All authors have read and agreed to the published version of the manuscript.

## FUNDING STATEMENT

Hayashibara Co., Ltd.

## CONFLICT OF INTEREST

S. E. and N. A. are employees of Hayashibara Co., Ltd. The company had no role in the design of the study; in the collection, analyses, or interpretation of data; in the writing of the manuscript, or in the decision to publish the results. All other authors declare no competing interests.

## Data Availability

Data can be made available on request.
